# Exploring the Expression and Perceived Relational Correlates of Perfectionism in Higher Education: A Multicenter Study

**DOI:** 10.3390/healthcare14060727

**Published:** 2026-03-12

**Authors:** Anna Marchetti, Anna De Benedictis, Elena Sandri, Valentina Micheluzzi, Michela Piredda, Maria Grazia De Marinis

**Affiliations:** 1 Research Unit Nursing Science, Department of Medicine and Surgery, University Campus Bio-Medico di Roma, Via Alvaro del Portillo, 21, 00128 Rome, Italy; a.marchetti@policlinicocampus.it (A.M.); a.debenedictis@policlinicocampus.it (A.D.B.); m.piredda@unicampus.it (M.P.); m.demarinis@unicampus.it (M.G.D.M.); 2 Research Unit of Nursing Palliative Care, Fondazione Policlinico Universitario Campus Bio-Medico, Via Alvaro del Portillo, 200, 00128 Rome, Italy; 3 Faculty of Medicine and Health Sciences, Catholic University of Valencia San Vicente Mártir, c/Quevedo, 2, 46001 Valencia, Spain; 4 Clinical and Interventional Cardiology, University Hospital of Sassari, 07100 Sassari, Italy; 5 Department of Medicine, Surgery and Pharmacy, University of Sassari, 07100 Sassari, Italy

**Keywords:** perfectionism, personality, mental health, sociodemographic factors, students, universities, Italy

## Abstract

**Highlights:**

**What are the main findings?**

- Perfectionism was mainly self-directed, with lower levels of perceived external expectations and demands toward others.
- Maladaptive perfectionism-related beliefs co-occurred with lower perceived trust/acceptance and stronger failure-based self-criticism, and the perceived external-pressure dimension showed the strongest links with Roots variables.

**What are the implications of the main findings?**

- University screening and prevention should go beyond a single overall score and prioritize perceived external evaluative pressure and self-critical responses to mistakes.
- Interventions may benefit from targeting tolerance of imperfection and adaptive mistake appraisal, using scalable delivery formats when appropriate.

**Abstract:**

**Background:** Perfectionism is a multidimensional disposition marked by exceptionally high standards and self-worth contingent on flawless performance. In university settings, academic demands may amplify perfectionistic pressure, with maladaptive outcomes most consistently linked to socially prescribed expectations and self-critical failure processing. This study profiled perfectionism dimensions in Italian university students and examined their associations with perceived relational and self-related correlates (Roots). **Methods:** A multicenter cross-sectional study was conducted with Italian university students. Participants completed two validated tools: the 14-item Multidimensional Perfectionism Scale—Revised (MPS-R) and the 16-item Roots questionnaire. Descriptive statistics, Spearman correlations, and non-parametric group comparisons were performed. **Results:** Self-oriented perfectionism was the most prominent dimension, while socially prescribed perfectionism (SPP) was comparatively lower but showed the clearest links with vulnerability-related correlates. Lower perceived parental and interpersonal trust was associated with stronger failure-based self-appraisals and perceived excessive demands from others. Higher SPP was observed among women and younger students and in more evaluative study contexts. **Conclusions:** Perfectionism in this sample was predominantly self-directed, yet risk-relevant profiles were characterized by SPP and self-critical failure processing in conjunction with lower perceived trust/acceptance. These findings support screening approaches that move beyond global scores and inform prevention strategies targeting fear of mistakes, contingent self-worth, and perceived evaluative pressure to promote student well-being. Longitudinal and intervention studies are needed to test temporal pathways and scalable, targeted prevention strategies.

## 1. Introduction

Perfectionism is a personality disposition involving the pursuit of exceptionally high standards and a self-evaluative style in which self-worth becomes contingent on flawless performance. In the influential model by Hewitt and Flett, perfectionism is articulated across three facets, namely self-oriented perfectionism (SOP), other-oriented perfectionism (OOP), and socially prescribed perfectionism (SPP), highlighting how perfectionistic demands may be directed toward the self, toward others, or experienced as imposed by the social environment [1]. Importantly, perfectionism is also increasingly salient in student populations: cross-temporal evidence indicates that SOP, OOP, and SPP have risen across cohorts of college students, underscoring its contemporary relevance in higher education [2].

Within this broader European context, recent evidence underscores the extent of emotional distress and vulnerability among university students, offering an important background for research on maladaptive perfectionism. For instance, a large cross-sectional survey in northern Italy reported widespread psychological distress and substantial anxiety/depressive symptoms, with differences by gender and academic stage [3]; consistent European findings further suggest that emerging adulthood in university is a high-risk period in which socioemotional and support-related factors shape vulnerability to anxiety-, depression-, and stress-related symptomatology [4,5,6,7]. Importantly, recent European research in nursing student populations, where evaluative pressure and emotional demands are often pronounced, has further highlighted burnout-related emotional burden and modifiable stress-related processes: an experimental study found that simulation-based learning experiences were associated with reductions in emotional exhaustion and cynicism and increases in perceived efficacy [8], while a cross-cultural comparison between Spain and Portugal documented meaningful differences in academic burnout alongside psychological and coping-related factors [9]. Taken together, these data reinforce the rationale for examining interpersonal and self-related correlates of perfectionism, particularly SPP, as a pathway through which perceived expectations and evaluative pressure may contribute to distress.

In university contexts, SOP reflects internally driven, rigid personal standards and an intense need to avoid mistakes [1]. SOP can align with disciplined goal pursuit and sustained effort; motivational models suggest that SOP may be linked to more self-determined forms of academic motivation, whereas SPP tends to relate to more controlled and externally pressured motivation [10]. However, the adaptiveness of SOP is conditional: meta-analytic evidence indicates that burnout risk is most consistently associated with perfectionistic concerns (e.g., harsh self-criticism, discrepancy-focused evaluation) rather than strivings alone [11]. In student samples, cognitive processes such as repetitive negative thinking (RNT), including worry and rumination, represent a key pathway connecting perfectionism dimensions to academic burnout facets (e.g., exhaustion, cynicism, inadequacy) [12]. Thus, SOP may be compatible with academic engagement when self-evaluation remains flexible but becomes detrimental when it is fused with rigid self-criticism and perseverative negative cognition [11,12].

SPP is characterized by the perception that others expect flawless performance and that one’s acceptance and approval are conditional on meeting these perceived external standards [1].

SPP is characterized by the belief that others require perfection and that acceptance depends on meeting these perceived external standards. Among the perfectionism facets, SPP has been repeatedly highlighted as a particularly detrimental form, showing robust links with distress and representing a public-health-relevant vulnerability factor [13]. In students, SPP is associated with lower academic self-efficacy and higher academic burnout, with evidence that academic self-efficacy can mediate the SPP–burnout relationship [14]. This pattern is consistent with the notion that perceived external demands undermine perceived competence and autonomy, thereby increasing emotional and motivational exhaustion [10,14].

OOP captures the tendency to demand perfection from other people, paired with critical evaluation when others fall short [1]. Contemporary evidence suggests that interpersonal forms of perfectionism contribute to relational strain and lower perceived quality of friendships and social connection, consistent with the Perfectionism Social Disconnection Model (PSDM) [15,16]. Domain-specific extensions indicate that perfectionistic expectations toward close others (e.g., partner-oriented forms) are associated with relational difficulties and show positive associations with narcissism-related features [17]. Overall, OOP-related demandingness may undermine collaborative functioning and relationship satisfaction via chronic dissatisfaction with others’ performance and conditional regard [1,17].

Beyond individual distress, perfectionism has a relational cost. The PSDM proposes that perfectionistic traits, especially SPP and perfectionistic self-presentation, erode perceived social support and promote loneliness, which in turn contributes to distress [15]. Recent evidence across undergraduate, law, and medical student samples supports this framework by demonstrating that loneliness mediates links between interpersonal perfectionism/perfectionistic self-presentation and distress, and that perfectionistic students tend to report lower social support [16]. In the Italian context, validated tools are available to assess the interpersonal expression of perfectionism (i.e., perfectionistic self-presentation), supporting more precise assessment of these socially relevant mechanisms [18].

Recent contributions have strengthened measurement and applied research on perfectionism in university settings in Italy. A multicenter study developed and psychometrically tested an inventory designed to assess not only manifestations but also perceived roots and perfectionism-related academic stress, offering a comprehensive tool for identifying risk-relevant profiles among students [19]. Another study has explored potentially modifiable correlates: humor styles explain incremental variance in perfectionism dimensions beyond personality traits, suggesting possible strengths-based targets [20]. Moreover, perfectionism-related features have been observed in student health-related phenotypes; in cross-cultural research including Italian university students, higher orthorexic tendencies were associated with higher perfectionism traits [21]. At the prevention level, Italian well-being programs for university students (e.g., NoiBene) explicitly include perfectionism and related transdiagnostic processes such as repetitive thinking among their outcomes, reflecting growing recognition of perfectionism as a modifiable risk process in student mental health promotion [22].

Despite these advances, evidence on how perfectionism is expressed and what students perceive as key relational and contextual correlates in university settings remains comparatively fragmented [11,12]. The present study addresses this gap by examining (a) the expression of SOP, OOP, and SPP in university students and (b) their associations with perceived relational/self correlates. This large-scale, multicenter study provides a characterization of perfectionism in Italian university students that combines the three core dimensions with perceived relational/self correlates. This integrated assessment helps move beyond global scores by highlighting heterogeneity in vulnerability patterns, thereby informing more targeted screening and prevention strategies in university mental-health promotion, with particular emphasis on reducing self-critical evaluation, persevering negative thinking, and social disconnection processes [12,13].

## 2. Materials and Methods

### 2.1. Study Design and Inclusion Criteria

This research was designed as a multicenter, cross-sectional observational study involving Italian university students. Eligible participants were undergraduate and master’s students, at least 18 years old, enrolled at Italian universities. Convenience sampling was conducted via institutional mailing lists from various Italian universities and through the investigators’ personal contacts. As a non-probabilistic approach, this sampling strategy may limit the representativeness of the sample and the generalizability of the findings to the overall Italian university student population.

### 2.2. Ethical Considerations

The study was conducted in accordance with the ethical principles of the Declaration of Helsinki [23] and received approval from the Ethics Committees of Fondazione Policlinico Universitario Campus Bio-Medico and Comitato Etico Territoriale Lazio Area 2 prior to initiation (Protocol FPUCBM 001.23(45.22) OSS19 April 2023 and 75.23CET2 cbm, 26 October 2023). Data protection and participant confidentiality were ensured in compliance with applicable data-protection regulations. All participants provided electronic informed consent for participation and data processing before completing and submitting the questionnaire.

### 2.3. Instruments

This study used two validated questionnaires. Perfectionism was assessed with the 14-item Multidimensional Perfectionism Scale—Revised (MPS-R), validated by Piredda et al. [19], a shortened version of the original 45-item scale developed by Hewitt et al. [1]. The MPS-R measures three dimensions: self-oriented (SOP), socially prescribed (SPP), and other-oriented perfectionism (OOP). The roots of perfectionism were measured with the 16-item Roots scale [19], which includes three factors: family relationships, relationship with the self, and social relationships. The Roots scale captures current subjective appraisals of relational and self-related experiences rather than objective childhood history or verified developmental antecedents; therefore, findings should be interpreted as perceived associations. Additional items collected sociodemographic information and other relevant data.

### 2.4. Data Collection

Data were collected through a multicenter dissemination strategy involving universities located in northern, central, and southern Italy, including the islands. The sample consisted of university students enrolled in undergraduate or master’s degree programs at participating institutions. This geographically diverse strategy aimed to capture students across academic levels and regions of Italy; however, the final sample composition reflects the recruitment channels used and includes an overrepresentation of students from health-science disciplines. The study adhered to the STROBE guidelines for observational research [24], and data were collected between January 2024 and March 2025.

### 2.5. Variables

#### 2.5.1. Multidimensional Perfectionism Scale—Revised (MPS-R)

The MPS-R consists of 14 items across three dimensions: self-oriented perfectionism (SOP; items 1, 3, 6, 9, and 12), other-oriented perfectionism (OOP; items 4, 7, 10, and 13), and socially prescribed perfectionism (SPP; items 2, 5, 8, 11, and 14). Items are rated on a 7-point Likert scale ranging from 1 (“strongly disagree”) to 7 (“strongly agree”). The total MPS-R score is obtained by summing responses to all 14 items (range 14–98). The scores of item 9 (I do not have to be the best at whatever I am doing) were reversed so that higher MPS-R scores indicate stronger perfectionistic tendencies [19]. The full list of MPS items is provided in the Supplementary File (Table S1).

For descriptive and interpretive purposes, Socially Prescribed Perfectionism (SPP) scores were classified into three categories: low (≤8), moderate (9–26), and high (≥27). These cut-off points correspond to the lower quartile, the central range, and the upper quartile of the theoretical score distribution, respectively. Although this categorization aids in the identification of risk-related profiles for screening and interpretative clarity, it may entail a loss of statistical power. Accordingly, all main inferential analyses were additionally conducted with SPP treated as a continuous variable to ensure the robustness and consistency of the results.

#### 2.5.2. 16-Item Roots Scale

The Roots scale comprises three dimensions: relationships with family (Family; items 1, 3, 4, 6, 8, 11, and 13), relationship with the self (Self; items 2, 5, 9, 12, 14, and 15), and social relationships (Social; items 7, 10, and 16). Each item is rated on a 7-point Likert scale from 1 (“strongly disagree”) to 7 (“strongly agree”). The overall score of Roots is computed by summing all the items (range 16–112). Items reflecting negative statements were reverse-scored so that Roots’ higher scores consistently indicate beliefs and perceptions that are protective toward maladaptive perfectionism. The full list of Roots items is provided in the Supplementary File (Table S2).

#### 2.5.3. Socio-Demographic Variables

The survey collected additional information on sociodemographic and academic variables, including sex, age, religion and religious practice, study area (e.g., health science, humanities, etc.) and level (i.e., bachelor’s or master’s degree), type of university (public or private), off-site study status, and scholarship or College of Merit placement. Age was grouped into young (18–24 years), young adults (25–39 years), and middle-aged (40–62 years). Students are classified as off-site when they move away from their home city to attend university, a circumstance that generally entails greater costs for housing and transportation. Colleges of Merit are residential institutions accredited by the Ministry of University and Research that select students through a highly competitive process emphasizing academic achievement and strong personal motivation; continued participation requires that students uphold these standards over time. Both being an off-site student and receiving a merit-based placement or scholarship can heighten the academic pressure students experience, as they may feel compelled to maintain exceptional performance.

### 2.6. Data Analysis

Prior to analysis, the dataset was pre-processed to exclude invalid, inconsistent, or extreme cases, including erroneous responses and statistical outliers. Missing data (non-responses) were addressed using listwise deletion, as each variable had less than 2% missing values—a proportion considered negligible for a sample of this size (*n* = 2103). Assessment of normality using the Shapiro–Wilk test, supported by visual inspection of Q–Q plots, showed that none of the variables were normally distributed [25].

Non-parametric tests (Mann–Whitney U and Kruskal–Wallis) were employed due to both the non-normal distribution of the data (Shapiro–Wilk *p* < 0.05) and the ordinal nature of the Likert-scale responses. Rank-based methods are therefore more appropriate and robust, helping to reduce the risk of Type I errors. Effect sizes were computed to evaluate practical significance, using the rank-biserial correlation (*r*) for Mann–Whitney U tests and epsilon-squared (*ε^2^*) for Kruskal–Wallis tests. Categorical variables are presented as frequencies and percentages, and continuous variables as means with standard deviations. A *p*-value < 0.05 was considered statistically significant. All statistical procedures were carried out using Jamovi software (version 2.6.28) [26], and supplementary visualizations were created and refined using Excel by Microsoft 365, version 2501, January 2025).

## 3. Results

### 3.1. Sample Description

A total of 2103 university students participated in the study (Table 1). The sample was predominantly female (76.2%), with a mean age of 23.4 years (SD = 5.68). Most participants were aged 18–24 years (76.46%), followed by 25–39 years (20.45%) and 40–62 years (3.09%).

Regarding religious affiliation, slightly more than half of the respondents identified as Catholic (52.4%). A substantial proportion reported being atheist or agnostic (27.2%), while 13.9% indicated religious indifference. Minority affiliations included Orthodox Christianity (1.9%), other Christian denominations (1.9%), Islam (0.8%), and other faiths. Only 28.9% of students reported actively practicing their religion. With respect to the academic field, most participants (70.2%) were enrolled in health sciences, followed by humanities (13.1%), engineering (8.3%), and sciences (5.8%). The distribution across academic years was balanced, with the largest proportions in the first year (23.2%) and second year (41.9%). Geographically, most participants came from central Italy (55.0%), followed by the main islands (21.9%), northern Italy (15.7%), and southern Italy (7.4%). Most participants attended public universities (81.6%), 35.8% were off-site students, and 19.4% received a scholarship or held a place in a College of Merit.

### 3.2. Analysis of MPS-R Responses

The item-level analysis of the Multidimensional Perfectionism Scale—Revised (MPS-R) reveals distinct patterns across its three dimensions:

Self-Oriented Perfectionism (SOP) (Figure S1a in Supplementary Material): This was the most prominent dimension. Approximately 80% of students endorsed high personal standards, and over 75% reported an inability to relax until tasks are perfect. Discomfort with errors was widespread, with 87.4% expressing some level of unease regarding mistakes.

Other-Oriented Perfectionism (OOP) (Figure S1b in Supplementary Material): Expectations directed toward others were present but less intense than self-directed ones. While nearly 50% held high expectations for significant others, strong agreement was less frequent for items regarding “top-notch quality” from others or intolerance of others’ mistakes.

Socially Prescribed Perfectionism (SPP) (Figure S1c in Supplementary Material): Perceived external pressure was moderate. The most frequent concern was the feeling that others are “too demanding” (26.1% agree), though only a minority (11.9%) explicitly linked their success to a need to please others.

**Figure 1:**
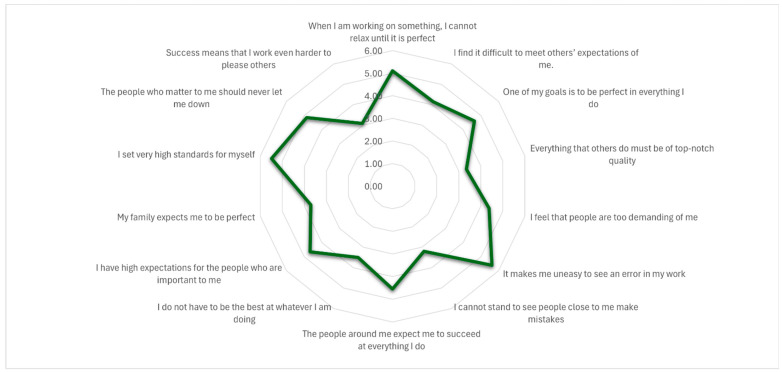
Graphical representation of individual item scores of MPS-R for the whole sample (N = 2103 university students).

Students in this sample primarily experience perfectionism as a self-imposed internal drive (SOP) rather than as a reaction to external social pressure (SPP) or demands placed on others (OOP). Table 2 reports descriptive statistics for each MPS-R item, the three perfectionism dimensions—self-oriented (SOP), other-oriented (OOP), and socially prescribed (SPP)—and the overall Multidimensional Perfectionism Scale—Revised (MPS-R) score. Figure 1 further illustrates the relative contribution of each dimension to overall perfectionism.

Overall, the findings indicate a clear tendency toward perfectionistic behavior in this sample. The highest mean was observed for “It makes me uneasy to see an error in my work” (M = 5.62, SD = 1.25), followed by “I set very high standards for myself” (M = 5.49) and “When I am working on something, I cannot relax until it is perfect” (M = 5.10). These results suggest pronounced self-imposed standards and discomfort with mistakes.

By contrast, items reflecting perceived external expectations were rated more moderately. For example, “The people who matter to me should never let me down” had a mean of 4.86, and “The people around me expect me to succeed at everything I do” scored 4.54. Perfectionistic demands directed toward others were comparatively lower, such as “Everything that others do must be of top-notch quality” (M = 3.35) and “I cannot stand to see people close to me make mistakes” (M = 3.20). Taken together, this pattern suggests that perfectionism in this sample was primarily self-directed rather than driven by external or interpersonal demands.

Across dimensions, SOP showed the highest mean (M = 4.86), followed by OOP (M = 4.03) and SPP (M = 3.98). The total MPS-R score was 60.3 (SD = 11.9), indicating a moderate-to-high level of perfectionistic tendencies among participants.

### 3.3. Analysis of Roots Responses

**Figure 2:**
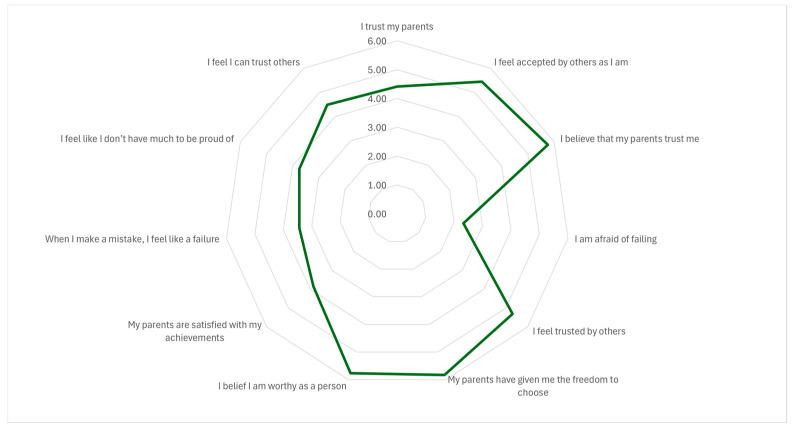
Graphical representation of Roots scores for the whole sample (N = 2103 university students).

The descriptive analysis of the Roots questionnaire (Table 3 and Figure 2) suggests that students generally perceived supportive and positive family dynamics. The highest mean scores were observed for items reflecting perceived affection, trust, and autonomy, including “I perceived myself as receiving love from my parents” (M = 5.94, SD = 1.42), “My parents have given me the freedom to choose in important matters” (M = 5.83, SD = 1.40), and “I believe that my parents trust me” (M = 5.76, SD = 1.42).

Regarding self-perceptions, participants reported relatively high scores on items related to personal worth, such as “I believe I am worthy as a person, regardless of my mistakes” (M = 5.77, SD = 1.36), while the reverse-coded item “No matter what I do, I feel inadequate” showed a more moderate mean (M = 4.05, SD = 1.80). Overall, this pattern is consistent with generally positive self-evaluations in the sample. However, the reverse-coded items “I am afraid of failing” (M = 2.33, SD = 1.60) and “When I make a mistake, I feel like a failure” (M = 3.44, SD = 1.89) indicate that fear of failure and self-critical reactions were present for a subset of participants.

Perceptions of social relationships were also broadly positive, with moderately high scores for “I feel accepted by others as I am” (M = 5.45, SD = 2.04) and “I feel I can trust others” (M = 4.50, SD = 1.87), suggesting a generally favorable sense of social acceptance and trust.

At the subscale level, Social Relationships showed the highest mean (M = 5.10, SD = 1.12), followed closely by Family Relationships (M = 5.06, SD = 1.01), while Self-Relationships had the lowest mean (M = 3.75, SD = 1.15). The overall Roots score averaged 73.20 (SD = 14.30; theoretical range 16–120), corresponding to a weighted mean of 4.58 (SD = 0.89; range 1–7).

In summary, students reported high levels of perceived parental love, trust, and autonomy, alongside generally positive self-evaluations. Nevertheless, comparatively lower scores on self-related items point to potential vulnerabilities in the self-domain, particularly around fear of failure and self-critical responses to mistakes, which may be relevant to the psychological foundations of perfectionistic traits.

The item-level analysis of the Roots scale (Figure S2a–c in Supplementary Material) reveals a predominantly positive relational background, albeit with specific areas of psychological vulnerability:

Family Relationships: Perceptions were overall highly positive. Most students reported strong parental love (51.1% maximum score) and high levels of autonomy and parental trust. However, explicit validation was less frequent, with only a small minority (10.2%) strongly feeling that their parents are satisfied with their achievements regardless of the outcome.

Relationship with the Self: This dimension displayed a dual pattern. While a significant group endorsed high self-worth (39.2%), this coexisted with high vulnerability; nearly half of the sample (44.6%) reported a maximum fear of failure, and approximately one-quarter linked mistakes directly to a sense of personal failure.

Social Relationships: Responses showed a high sense of social acceptance (48.5% maximum score) and being trusted by others. However, students’ reciprocal trust toward others was more cautious, showing a more even distribution across intermediate values rather than clustering at the highest scores.

The sample is characterized by strong perceived familial support and social acceptance, yet it remains highly susceptible to performance-based self-criticism and a pronounced fear of failure.

### 3.4. Associations Between Manifestations and Roots of Perfectionism

**Figure 3:**
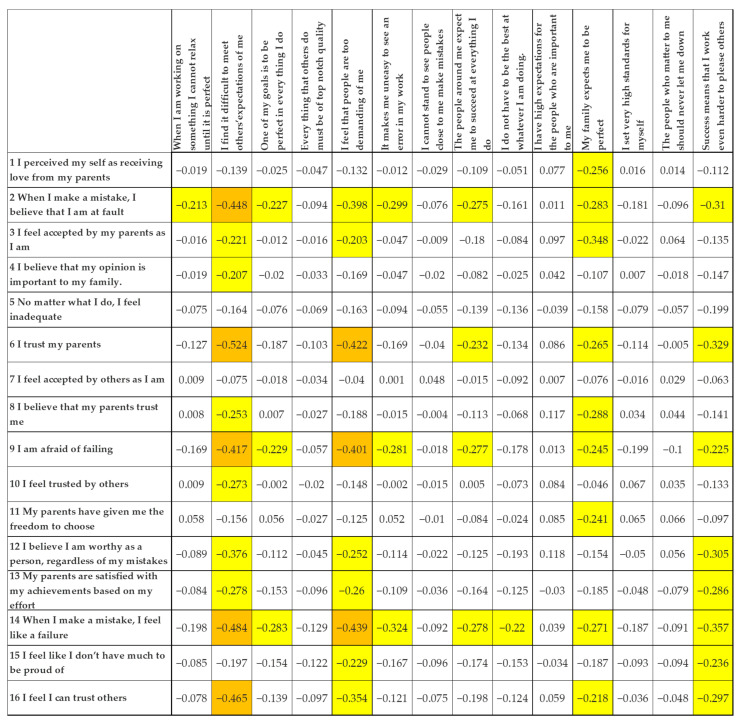
Spearman’s correlation analysis between dimensions of perfectionism and the roots of perfectionism. Legend: White cell = no correlation (0.00–0.19); yellow cell = low correlation (0.20–0.39); orange cell = moderate correlation (0.40–0.59). (NOTE: Only the correlations between the variables from the Multidimensional Perfectionism Scale—Revised (MPS-R) and variables from the Roots are reported. Correlations between variables within the same scale have been omitted, as these items are expected to be interrelated by design, given that they collectively constitute a psychometric scale).

The correlation matrix in Figure 3 examines associations between perfectionism components measured by the MPS-R and a set of psychological, familial, and self-perception variables that may be related to perfectionism-relevant experiences. Overall, the pattern is coherent, particularly in how perceived parental support and interpersonal trust negatively relate to more maladaptive perfectionistic beliefs.

The strongest negative correlations (orange cells) consistently highlight links between perceived trust/acceptance and maladaptive perfectionism-related cognitions. For example, perceiving one’s parents as trustworthy was strongly and negatively associated with the belief that others are too demanding (r = −0.524) and with endorsing the idea that making mistakes implies being a failure (r = −0.484). Similarly, believing that one can trust others was negatively related to feeling like a failure (r = −0.465) and to perceiving others as excessively demanding (r = −0.417). Taken together, these associations indicate that lower perceived trust and acceptance co-occur with a more self-critical and punitive interpretation of mistakes.

Moderate correlations (yellow cells) further support this pattern. Indicators of acceptance and perceived support, such as feeling accepted by parents (r = −0.348), perceiving that parents are satisfied with one’s achievements (r = −0.286), and reporting freedom of choice (r = −0.241), tended to be lower among students reporting stronger perfectionistic beliefs. In addition, self-blame and negative self-appraisals were negatively associated with parental recognition and trust (e.g., r = −0.283 between “When I make a mistake, I believe that I am at fault” and “I feel accepted by my parents as I am”).

Overall, the findings suggest that maladaptive perfectionism-related beliefs are associated with lower perceived trust in others and weaker perceptions of unconditional acceptance within the family context. While the strongest correlations point to a close link between low trust/acceptance and intense self-criticism, the moderate associations indicate a broader pattern in which perceived pressure, fear of failure, and lower self-worth tend to co-occur.

### 3.5. Analysis of Socially Prescribed Perfectionism (SPP) According to Sociodemographic Variables

As previously observed, the Socially Prescribed Perfectionism (SPP) dimension shows the strongest associations with the Roots variables. For this reason, it was chosen for more in-depth analysis. Based on the SPP scores reported by university students, the sample was divided into three categories:

- Low SPP, corresponding to scores ≤8 (representing the lowest 25% of the possible SPP range, which spans from 0 to 35);
- Moderate SPP, including scores from 9 to 26;
- High SPP, corresponding to scores ≥27 (representing the top 25% of the possible range).

**Figure 4:**
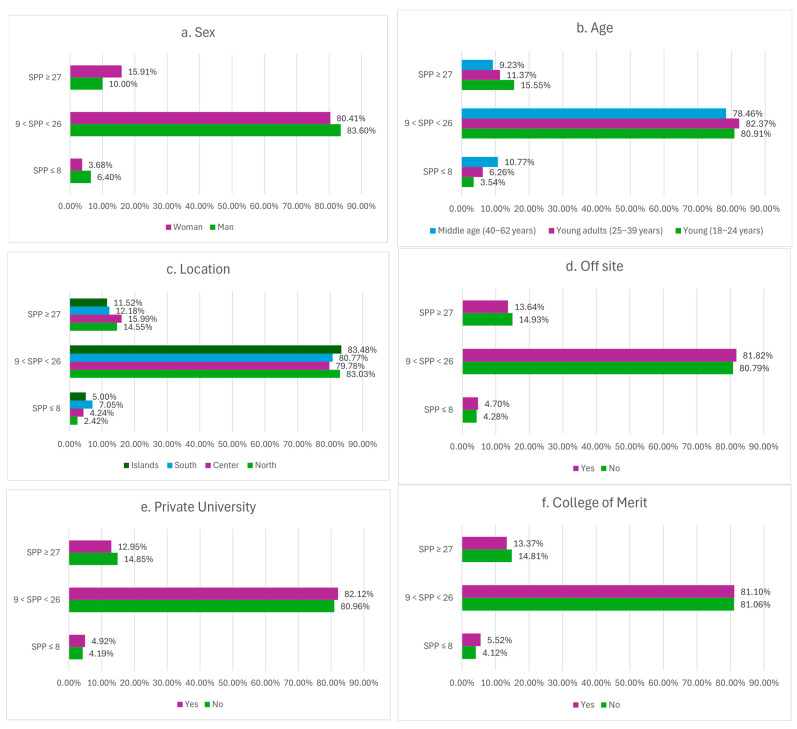

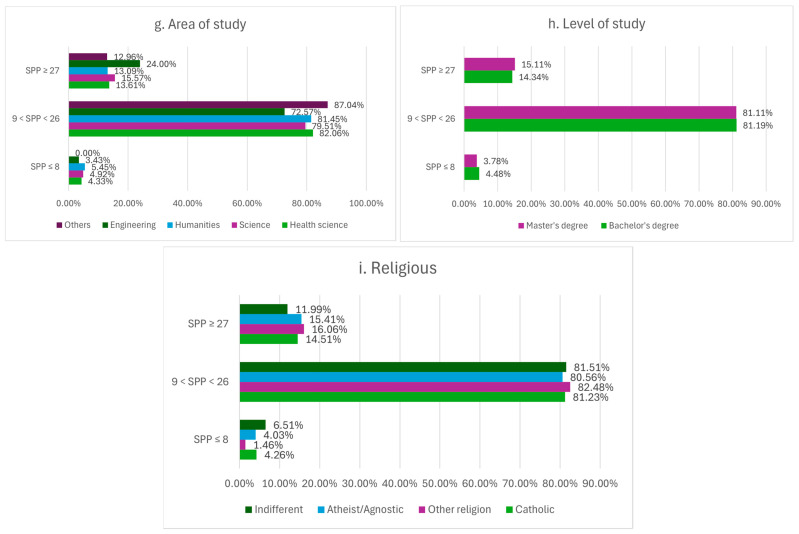
(**a**–**i**). Distribution of sociodemographic variables across three SPP levels. Note: SPP = Socially Prescribed Perfectionism. The percentages shown are relative percentages, computed by dividing the number of participants who gave a response by the total number of individuals within that specific category.

Subsequently, participants’ distribution across these three SPP levels was examined in relation to sociodemographic variables to explore whether any factors were associated with socially prescribed perfectionism. Detailed results are presented in Figure 4a–i.

Overall, female participants reported slightly higher SPP levels than males, suggesting greater sensitivity to perceived external expectations and social pressure, in line with previous studies. SPP levels also tended to be higher in younger age groups, with a gradual decrease as age increased, indicating that younger students may be more susceptible to perceived social demands.

Regional differences were observed, with students from northern and central Italy generally reporting higher SPP than those from southern regions and the islands. On-site students reported higher SPP than off-site students, defined as those who relocate to another city to pursue their studies. This difference may reflect the additional financial burden and adaptation-related stress associated with living away from home, as well as distinct social and academic demands compared with students who remain in their place of origin.

Students enrolled in private universities showed slightly higher SPP levels than those in public institutions. The distribution of SPP scores was similar between students attending a College of Merit and those who did not. In both groups, most participants fell within the intermediate SPP range (9 ≤ SPP < 26), accounting for approximately 81%. Low (SPP ≤ 8) and high (SPP ≥ 27) scores were less frequent, with only minor differences between the two groups. Undergraduate students tended to score higher on SPP than graduate students, suggesting that earlier stages of higher education may involve more intense experiences of external evaluation and social comparison, which may attenuate with increasing academic maturity and autonomy.

SPP also varied across academic disciplines. For instance, health sciences and law showed higher SPP than other disciplines, although differences were not uniformly large. Finally, SPP differed only modestly across religious categories, with most participants in each group falling in the mid-range and small differences emerging primarily at the extremes.

To assess the practical relevance of sociodemographic differences, effect sizes were computed for all group comparisons (see Tables S3 and S4, Supplementary file). Although women reported higher levels of SPP than men, the magnitude of this difference was small (r = 0.130, *p* < 0.001). Likewise, variations in SPP across age groups reached statistical significance but reflected a minimal practical effect (ε^2^ = 0.008, *p* < 0.001). Differences among academic disciplines were also statistically significant; however, the associated effect size was negligible (ε^2^ = 0.003, *p* = 0.042). Overall, these findings indicate that while sociodemographic characteristics are related to perceived external pressure, their individual impact is modest when compared with the influence of relational and self-related factors identified in the multivariable analyses.

### 3.6. Hierarchical Regression Analysis: Predicting Socially Prescribed Perfectionism Through “Roots”

A hierarchical multivariable linear regression analysis was performed to evaluate the distinct and incremental contribution of perceived self-related and relational “Roots” to Socially Prescribed Perfectionism (SPP), while adjusting for sociodemographic variables and other dimensions of perfectionism, namely Self-Oriented Perfectionism (SOP) and Other-Oriented Perfectionism (OOP) (Table 4). In the first step, sociodemographic factors together with SOP and OOP were entered into the model, accounting for 18.0% of the variance in SPP (*R*^2^ = 0.180, *p* < 0.001). In the second step, the three Roots subscales were included, leading to a statistically significant improvement in model fit (*ΔR*^2^ = 0.255, *F*(3, 2090) = 315, *p* < 0.001). The final model explained 43.5% of the total variance in SPP (*R*^2^ = 0.435).

In the final model, all three Roots dimensions emerged as significant independent negative predictors of SPP. The strongest effects were observed for Relationship with the Self (*β* = −0.285, *p* < 0.001) and Relationships with Family (*β* = −0.260, *p* < 0.001), followed by Social Relationships (*β* = −0.096, *p* < 0.001). These results indicate that higher levels of perceived trust, acceptance, and self-worth are strongly and uniquely associated with lower perceived external evaluative pressure, even after controlling for internal perfectionistic tendencies (SOP) and externally directed perfectionistic demands (OOP).

## 4. Discussion

In this large, multicenter cross-sectional study, we investigated how the main dimensions of perfectionism (SOP, OOP, and SPP) are expressed among university students and how they are associated with self-reported relational and self-related experiences (Roots), as well as relevant psychological, academic, and interpersonal correlates.

The sample comprised 2103 students and was predominantly female (76.2%). The predominance of female students in the present sample should be interpreted not only as a limitation in terms of gender balance but also as a structural feature of Italian higher education. National enrollment data indicate a higher proportion of women across university programs (e.g., 56% in bachelor’s programs, 67% in master’s programs, and approximately 76% in nursing) [27]. This composition has important implications for inclusivity and differential vulnerability. Female students and those enrolled in health-related disciplines may be exposed to heightened relational, evaluative, and caregiving expectations, which can interact with perfectionistic concerns and fear of failure [28,29]. Consequently, the expression and perceived relational correlates of perfectionism observed in this study may be especially salient for these groups. At the same time, the underrepresentation of male students and non-health-science disciplines suggests that future research should further investigate how perfectionism is expressed and experienced across more diverse academic fields, gender identities, and sociocultural contexts.

Participants had a mean age of 23.4 years (SD = 5.68). Most were aged 18–24 years (76.46%), followed by 25–39 years (20.45%) and 40–62 years (3.09%). Most participants were enrolled in bachelor’s programs (78.6%) and attended public universities (81.6%); 35.8% were off-site students, and 19.4% received a scholarship or held a place in a College of Merit.

Although the Roots scale does not assess objective developmental antecedents, perceived relational climates remain theoretically and clinically meaningful constructs. Subjective appraisals of parental expectations, relational dynamics, and self-evaluative standards are known to shape individuals’ current self-concept, emotional responses, and interpersonal functioning. In the context of perfectionism, how students perceive relational expectations and evaluative pressures may be particularly relevant, as these perceptions can influence ongoing patterns of self-criticism, fear of negative evaluation, and relational sensitivity. Accordingly, the present findings should be interpreted as reflecting perceived relational correlates of perfectionism rather than verified developmental pathways, while still offering valuable insight into students’ lived psychological experiences.

SOP was the predominant dimension, whereas SPP and OOP were lower. Overall, the profile suggests that perfectionism was primarily experienced as an internal, self-imposed drive, whereas vulnerability-relevant patterns were more closely aligned with SPP and self-critical, failure-related processing. However, the Roots profile was not uniformly protective: although students generally reported high perceived parental love, trust, and autonomy, and relatively positive social acceptance, a subset also reported self-domain vulnerabilities, especially fear of failure and failure-based self-evaluations, together with more maladaptive perfectionism-related beliefs.

These findings align with contemporary evidence indicating that perfectionism is multidimensional and that perfectionistic concerns (e.g., fear of negative evaluation, self-criticism, discrepancy-focused evaluation) tend to be more strongly associated with psychological distress than perfectionistic strivings (e.g., high personal standards) [30]. Even when SOP is high, risk increases when high standards are accompanied by perfectionistic concerns, such as fear of mistakes/failure sensitivity and contingent self-worth, which are consistently associated with anxiety, depressive, and obsessive–compulsive symptoms in adolescents and emerging adults [31,32,33]. Accordingly, our findings are best interpreted as reflecting heterogeneity: similar high standards may coexist with markedly different levels of error sensitivity and failure-based self-appraisal, which are more proximally linked to maladaptive outcomes.

Interpreted through the lens of the Perfectionism Social Disconnection Model (PSDM), the Roots findings provide complementary context for understanding the observed patterns of perfectionism. Overall, the Family and Social Relationships subscales showed the highest mean scores, suggesting that students generally perceived environments as supportive. In contrast, the Relationship with the Self subscale yielded the lowest score, and distributional analyses indicated that a substantial subset of students endorsed fear of failure and self-critical responses to mistakes [19,33,34].

Consistent with PSDM-informed interpretations, correlation analyses showed that lower perceived parental trust and acceptance, as well as lower generalized interpersonal trust, were strongly associated with failure-based self-appraisals and the perception that others are excessively demanding [16,35].

Although these cross-sectional associations do not allow causal or directional inferences, they indicate a co-occurrence between perceived relational insecurity and more punitive interpretations of mistakes alongside heightened perceived external pressure [36].

Within the PSDM framework, such patterns are theoretically meaningful, as concerns about evaluation and reduced interpersonal security are thought to reinforce self-critical perfectionism and avoidance of imperfection in social contexts [15,16].

Notably, while items reflecting social acceptance were generally rated highly, trust in others showed a more cautious pattern, with responses clustering in the intermediate range. This nuance suggests that feeling accepted may not necessarily translate into feeling sufficiently safe to rely on others, disclose imperfections, or tolerate mistakes within interpersonal relationships—processes that are central to PSDM accounts of social disconnection and vulnerability to distress [37].

With respect to demographic and contextual factors, we observed higher SPP among women than men and higher SPP in younger students, with a gradual decrease across age groups. This pattern is broadly consistent with evidence linking SPP to heightened evaluative concerns (e.g., fear of negative evaluation) and suggesting that socially prescribed pressures may be particularly salient among female students and in emerging adulthood [38,39,40]. In this context, socially prescribed pressures may be differentially internalized through gendered socialization and evaluative norms, potentially increasing sensitivity to perceived expectations and external standards. However, these sociocultural explanations are tentative and should be interpreted as plausible correlates rather than causal mechanisms [41]. Moreover, these demographic differences, while statistically significant, showed small effect sizes, suggesting that individual psychological and relational backgrounds (the ‘roots’) may play a more prominent role. We also observed regional differences, with higher SPP in Northern/Central Italy than in the South/Islands, suggesting that greater exposure to peer comparison and institutional evaluation may be associated with stronger perceived external demands [42,43]. This disparity might reflect the distinct socio-economic climates and institutional cultures across Italian regions, where Northern and Central universities often embody higher levels of perceived competitiveness and meritocratic evaluation. In addition, slightly higher SPP levels in private universities may reflect more competitive academic climates and stronger norms of excellence [13,43]. Finally, undergraduates tended to report higher SPP than graduate students, consistent with the possibility that perceived social evaluation and uncertainty are most pronounced in earlier stages of academic training [19]. As students progress, increasing academic maturity and autonomy may act as buffers, attenuating the sensitivity to external evaluation. Beyond evaluative concerns, SPP may also be linked to burnout risk, particularly emotional exhaustion, in higher education and professional training contexts. Perceived external demands and fear of negative evaluation may foster persistent overinvestment and reduced recovery, contributing to exhaustion over time. Recent European studies in nursing students support the relevance of burnout-related outcomes in this population and point to the potential protective role of emotional and coping resources [8,9]. Within a PSDM-informed perspective, these patterns further suggest that perceived evaluative pressure may operate alongside interpersonal insecurity to amplify strain and exhaustion-related outcomes, even when overall perfectionism appears predominantly self-directed.

Discipline-level differences also emerged, with higher SPP in fields commonly associated with high professional stakes and intensive evaluation (e.g., health sciences, law), although the magnitude of these differences was modest. This pattern is consistent with reports of elevated perfectionism, particularly concern-related facets, in some professional training contexts [44]. However, evidence of variability across institutions and cohorts cautions against assuming uniform profiles across programs and time periods, as selection and enculturation processes may differ by educational setting [43]. From a measurement perspective, combining the MPS-R with Roots (i.e., perceived relational and self-related correlates) aligns with previous Italian psychometric work emphasizing culturally adapted assessment and the relevance of interpersonal facets for identifying risk-relevant profiles [19,45].

Taken together, our results suggest that relying solely on a global perfectionism score may obscure meaningful heterogeneity: students may endorse similarly high standards yet differ substantially in fear of failure, interpersonal trust, and perceived unconditional acceptance, factors that were closely aligned with maladaptive perfectionism-related cognitions. In line with the PSDM, this supports interpreting maladaptive perfectionism not only as an intrapersonal standard-setting style but also as a relationally embedded pattern shaped by perceived acceptance, trust, and evaluative concerns.

### Implications and Future Directions

Clinical and educational implications point to moving beyond a unitary view of “perfectionism” and targeting specific mechanisms, particularly socially prescribed evaluative pressure (SPP) and failure-centered self-critical cognitions, which are consistently linked to psychological distress [13]. In parallel, the co-occurrence of high self-oriented perfectionism (SOP) and marked error sensitivity supports interventions that build tolerance of imperfection and reframe mistakes as learning-relevant information rather than as threats to self-worth [33]. 

At the institutional level (i.e., university teaching and assessment practices), structural strategies, such as transparent grading criteria/rubrics and feedback practices that normalize errors, may help prevent self-imposed standards from escalating into dysfunctional self-criticism, especially in high-evaluation contexts [46]. In this framework, digital and scalable interventions are particularly relevant: internet-based CBT for perfectionism (iCBT-P) has accumulating evidence for reducing perfectionism and related symptoms, with benefits maintained at follow-up, making it suitable for university services and stepped-care models [47]. 

Mechanistically, programs that combine graded exposure to imperfection with cognitive restructuring of contingent self-worth and rigid standards are consistent with this risk profile; moreover, PSDM-informed components, such as reducing perfectionistic self-presentation and facilitating gradual disclosure of “non-perfect” performances, may be especially helpful when perfectionism co-occurs with low interpersonal trust and high perceived demands [37].

Future research should test process models examining whether error reactivity (e.g., shame, threat appraisals, rumination/worry) mediates links between SPP and functional outcomes (avoidance, procrastination, burnout), and evaluate the reach and acceptability of tailored digital programs in university settings for subgroups with higher SPP. Longitudinal designs will be crucial to clarify directionality among perceived relational climate, trust, fear of failure, and perfectionism trajectories across the university years.

In interpreting the present findings, several methodological strengths and limitations should be considered. Strengths include the large multicenter sample with broad geographic national coverage and the use of validated instruments, allowing concurrent assessment of perfectionism dimensions and perceived relational and self-related correlates. Stratification by socially prescribed perfectionism further enabled the identification of sociodemographic patterns associated with perceived evaluative pressure.

Limitations include the use of convenience sampling and an imbalanced sample composition characterized by a predominance of female students and a high representation of health sciences disciplines. As acknowledged in the Methods, these features may introduce selection bias and limit the generalizability of the findings to the broader Italian university student population. In particular, the overrepresentation of women and health-related disciplines may have influenced the observed profile of perfectionism and its perceived correlates, warranting caution when extending these results to male students or to other academic fields. At the same time, this composition reflects structural characteristics of Italian higher education, especially within healthcare programs [27], which should be considered when interpreting the relevance of the findings in context.

Exclusive reliance on self-report measures increases susceptibility to recall and social desirability biases and to shared-method variance, potentially inflating observed associations. In addition, Roots captures perceived relational and self-related correlates rather than prospective developmental antecedents; therefore, interpretations in terms of “origins” should be made cautiously. Finally, categorizing SPP into groups (low/moderate/high), while useful for interpretation and visualization, may reduce statistical power and obscure within-group variability compared with analyses treating SPP as a continuous construct.

## 5. Conclusions

This multicenter cross-sectional study provides a comprehensive characterization of perfectionism in Italian university students by integrating dimensional assessment (MPS-R) with perceived relational/self correlates (Roots). The findings indicate a predominantly self-directed perfectionism profile, while suggesting that clinically relevant vulnerability is more closely associated with socially prescribed evaluative pressure and self-critical failure processing. These results emphasize the value of assessing perfectionism beyond global scores and support the view that prevention in university settings should target mechanisms such as fear of mistakes, contingent self-worth, and perceived lack of unconditional acceptance. Importantly, these markers may also support early identification of students at heightened risk of emotional distress and burnout-related outcomes (e.g., emotional exhaustion), enabling timely referral and preventive support within university services. Future longitudinal and intervention studies are needed to clarify temporal relationships and to test scalable, targeted approaches for students most vulnerable to maladaptive perfectionism.

## Figures and Tables

**Table 1:** Sample and their socio-demographic characteristics (N = 2103 university students).

Sex	N	%
Male	500	23.80%
Female	1603	76.20%
Age	Mean	(SD)
	23.4	5.68
	N	%
Young (18–24 years)	1608	76.46%
Young adults (25–39 years)	431	20.45%
Middle age (40–62 years)	65	3.09%
Study level	N	**%**
Bachelor’s degree	1653	78.60%
Master’s degree	450	21.40%
Study field	N	%
Health science (Nursing, Medicine, Dentistry, Nutrition, Psychology)	1477	70.20%
Science (Mathematics, Physics, Chemistry, Biology, Biotechnology, etc.)	122	5.80%
Humanities (Philosophy, Literature, Communication, Law, Educational sciences, Economics, etc.)	275	13.10%
Engineering	175	8.30%
Other	54	2.60%
Course year	N	%
1	488	23.20%
2	881	41.90%
3	396	18.80%
4	120	5.70%
5	147	7.00%
6	71	3.40%
Geographical area	N	%
North	330	15.70%
Center	1157	55.00%
South	156	7.40%
Main islands	460	21.90%
Private University	N	%
No	1717	81.60%
Yes	386	18.40%
Off-site	N	%
No	1145	64.18%
Yes	638	35.76%
No answer	320	17.94%
Scholarship/place in a College of Merit	N	%
No	1431	80.62%
Yes	344	19.38%
No answer	328	18.48%
Religious faith	N	%
Roman catholic	1103	52.40%
Other religion	137	6.50%
Atheist/Agnostic	571	27.20%
Indifferent	292	13.90%
Religious practice	N	%
No	1496	71.10%
Yes	607	28.90%

**Table 2:** Scores on the MPS-R questionnaire for the whole sample (N = 2103 university students).

	All Sample			
	Mean	SD		
When I am working on something, I cannot relax until it is perfect	5.10	1.33		
I find it difficult to meet others’ expectations of me	4.14	1.70		
One of my goals is to be perfect in everything I do	4.63	1.71		
Everything that others do must be of top-notch quality	3.35	1.50		
I feel that people are too demanding of me	4.38	1.68		
It makes me uneasy to see an error in my work	5.62	1.25		
I cannot stand to see people close to me make mistakes	3.20	1.59		
The people around me expect me to succeed at everything I do	4.54	1.66		
I do not have to be the best at whatever I am doing	3.50	1.75		
I have high expectations for the people who are important to me	4.66	1.45		
My family expects me to be perfect	3.71	1.87		
I set very high standards for myself	5.49	1.47		
The people who matter to me should never let me down	4.86	1.60		
Success means that I work even harder to please others	3.08	1.86		
SOP (Self-oriented perfectionism)	24.30	5.37	4.86 ^	1.07 ^
OOP (Other-oriented perfectionism)	16.10	4.36	4.03 ^	1.09 ^
SPP (Socially prescribed perfectionism)	19.90	6.23	3.98 ^	1.56 ^
MPS-R	60.30	11.90	4.31 ^	0.85 ^

^ Weighted value for the number of items in each dimension.

**Table 3:** Roots scores for the whole sample (N = 2103 university students).

	Mean	SD		
I perceived myself as receiving love from my parents	5.94	1.42		
When I make a mistake, I believe that I am at fault	3.18	1.71		
I feel accepted by my parents as I am	5.52	1.65		
I believe that my opinion is important to my family	4.12	1.92		
No matter what I do, I feel inadequate	4.05	1.80		
I trust my parents	4.41	1.91		
I feel accepted by others as I am	5.45	2.04		
I believe that my parents trust me	5.76	1.42		
I am afraid of failing	2.33	1.60		
I feel trusted by others	5.31	1.19		
My parents have given me the freedom to choose	5.83	1.40		
I believe I am worthy as a person	5.77	1.36		
My parents are satisfied with my achievements	3.85	1.78		
When I make a mistake, I feel like a failure	3.44	1.89		
I feel like I don’t have much to be proud of	3.74	1.80		
I feel I can trust others	4.50	1.87		
RELATIONSHIPS WITH FAMILY	35.40	7.06	5.06 ^	1.01 ^
RELATIONSHIPS WITH THE SELF	22.50	6.89	3.75 ^	1.15 ^
SOCIAL RELATIONSHIPS	15.30	3.37	5.10 ^	1.12 ^
ROOTS	73.20	14.30	4.58 ^	0.89 ^

^ Weighted value for the number of items in each dimension.

**Table 4:** Hierarchical linear regression analysis predicting Socially Prescribed Perfectionism (SPP) (N = 2103).

Predictor	Estimator	SE	t	*p*	Standard Estimator
Constant	28.616	0.922	31.020	<0.001	
Sex	0.309	0.250	1.240	0.216	0.021
Age	−0.420	0.209	−2.010	0.045	−0.034
Religious	−0.455	0.092	−4.980	<0.001	−0.085
Course of Studies	−0.074	0.055	−1.350	0.177	−0.033
Area of study	0.154	0.134	1.150	0.251	0.028
Location	−0.218	0.110	−1.980	0.048	−0.035
Private University	−0.325	0.313	−1.040	0.298	−0.020
SOP	0.225	0.022	10.240	<0.001	0.194
OOP	0.195	0.026	7.650	<0.001	0.137
RELATIONSHIPS WITH FAMILY	−0.230	0.019	−11.850	<0.001	−0.260
RELATIONSHIPS WITH THE SELF	−0.257	0.021	−12.380	<0.001	−0.285
SOCIAL RELATIONSHIPS	−0.177	0.041	−4.350	<0.001	−0.096

Note: In Block 1, sociodemographic variables together with self-oriented and other-oriented perfectionism were entered into the model. Block 2 subsequently included the Roots subscales. SE denotes standard error; t refers to the *t*-test statistic; *p* indicates the *p*-value; β represents the standardized coefficient. Socially Prescribed Perfectionism (SPP) was assessed using the MPS-R, while the Roots dimensions were measured with the Roots scale.

## Data Availability

The data presented in this study are available from the corresponding author upon reasonable request. The data are not publicly available due to privacy and ethical restrictions.

## Supplementary Materials

The following supporting information can be downloaded at: https://www.mdpi.com/article/10.3390/healthcare14060727/s1, Figure S1. a Distribution of responses for the SOP dimension of the MPS-R scale; Figure S1. b Distribution of responses for the OOP dimension of the MPS-R scale; Figure S1. c Distribution of responses for the SPP dimension of the MPS-R scale; Figure S2. a. Distribution of responses for the dimensions of roots of perfectionism: Relationships with family; Figure S2. b. Distribution of responses for the dimensions of roots of perfectionism: Relationships with the self; Figure S2. c Distribution of responses for the dimensions of roots of perfectionism: Social relationships; Table S1: Items of the Multidimensional Perfectionism Scale—Revised (MPS-R); Table S2. Item of the Roots scale; Table S3. Sensitivity Analysis: Comparison of Results between Categorical (Group-based) and Continuous (Regression-based) Approaches for Socially Prescribed Perfectionism (SPP); Table S4. Effect sizes for group comparisons of Socially Prescribed Perfectionism (SPP).

## Abbreviations

The following abbreviations are used in this manuscript:

ANVUR	National Agency for the Evaluation of Universities and Research Institutes
CBT	Cognitive Behavioral Therapy
iCBT-P	Internet-based Cognitive Behavioral Therapy for Perfectionism
MPS-R	Multidimensional Perfectionism Scale—Revised
ROOTS	Roots questionnaire/scale
SOP	Self-oriented perfectionism
SPP	Socially prescribed perfectionism
OOP	other-oriented perfectionism
PSDM	Perfectionism Social Disconnection Model
RNT	Repetitive Negative Thinking
STROBE	Strengthening the Reporting of Observational Studies in Epidemiology
SD	Standard Deviation
