# Supplementary material for: Exploring the Expression and Perceived Relational Correlates of Perfectionism in Higher Education: A Multicenter Study
Source: Healthcare (Basel). 2026 Mar 12;14(6):727. doi: 10.3390/healthcare14060727 (PMC13026897; doi:10.3390/healthcare14060727)
Supplement: Supplementary file 1 [file healthcare-14-00727-s001.zip › healthcare-4087538-supplementary.pdf]

Supplementary Material

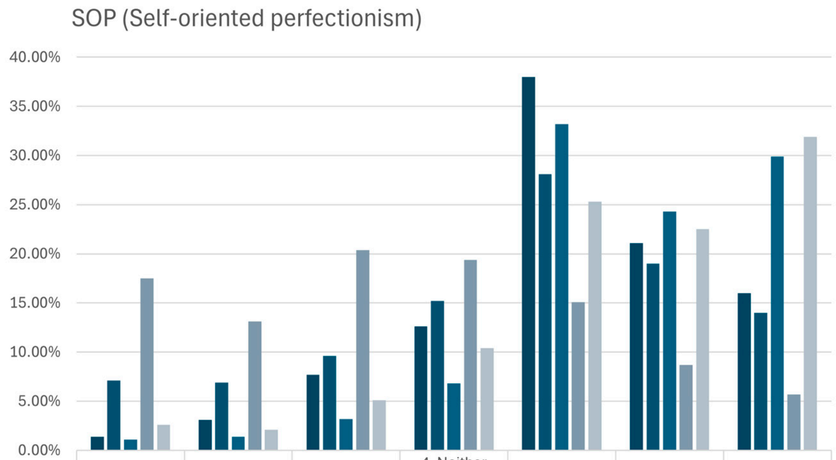

	1. Strongly disagree	2. Disagree	3. Somewhat disagree	4. Neither agree nor disagree	5. Somewhat agree	6. Agree	7. Strongly agree
When I am working on something, I cannot relax until it is perfect %	1.40%	3.10%	7.70%	12.60%	38.00%	21.10%	16.00%
One of my goals is to be perfect in everything I do %	7.10%	6.90%	9.60%	15.20%	28.10%	19.00%	14.00%
It makes me uneasy to see an error in my work %	1.10%	1.40%	3.20%	6.80%	33.20%	24.30%	29.90%
I do not have to be the best at whatever I am doing %	17.50%	13.10%	20.40%	19.40%	15.10%	8.70%	5.70%
I set very high standards for myself %	2.60%	2.10%	5.10%	10.40%	25.30%	22.50%	31.90%

Figure S1. a Distribution of responses for the SOP dimension of the MPS-R scale.

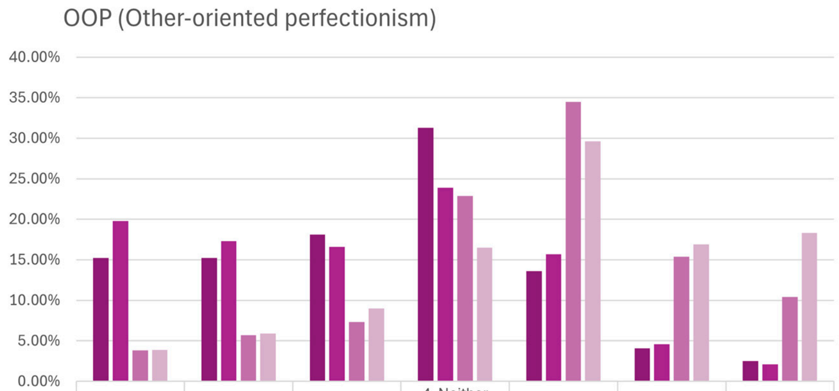

	1. Strongly disagree	2. Disagree	3. Somewhat disagree	4. Neither agree nor disagree	5. Somewhat agree	6. Agree	7. Strongly agree
Everything that others do must be of top-notch quality %	15.20%	15.20%	18.10%	31.30%	13.60%	4.10%	2.50%
I cannot stand to see people close to me make mistakes %	19.80%	17.30%	16.60%	23.90%	15.70%	4.60%	2.10%
I have high expectations for the people who are important to me %	3.80%	5.70%	7.30%	22.90%	34.50%	15.40%	10.40%
The people who matter to me should never let me down %	3.90%	5.90%	9.00%	16.50%	29.60%	16.90%	18.30%

Figure S1. b Distribution of responses for the OOP dimension of the MPS-R scale.

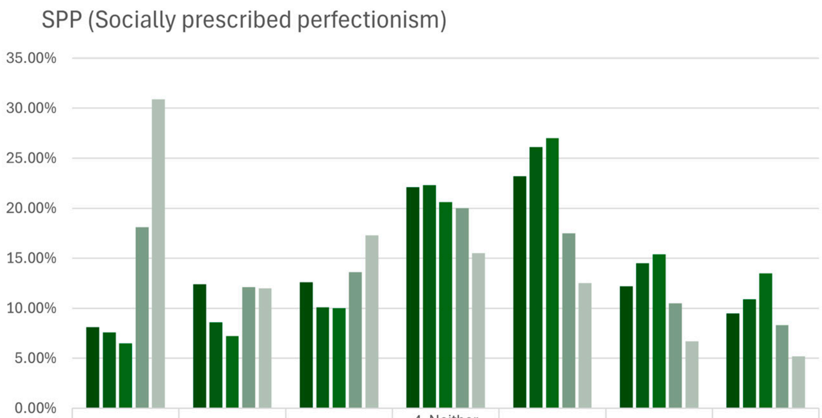

	1. Strongly disagree	2. Disagree	3. Somewhat disagree	4. Neither agree nor disagree	5. Somewhat agree	6. Agree	7. Strongly agree
I find it difficult to meet others' expectations of me %	8.10%	12.40%	12.60%	22.10%	23.20%	12.20%	9.50%
I feel that people are too demanding of me %	7.60%	8.60%	10.10%	22.30%	26.10%	14.50%	10.90%
The people around me expect me to succeed at everything I do %	6.50%	7.20%	10.00%	20.60%	27.00%	15.40%	13.50%
My family expects me to be perfect %	18.10%	12.10%	13.60%	20.00%	17.50%	10.50%	8.30%
Success means that I work even harder to please others %	30.90%	12.00%	17.30%	15.50%	12.50%	6.70%	5.20%

Figure S1. c Distribution of responses for the SPP dimension of the MPS-R scale.

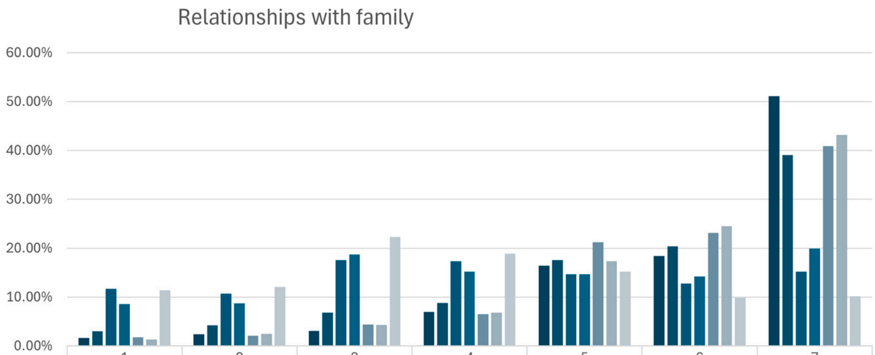

	1	2	3	4	5	6	7
I perceived myself as receiving love from my parents	1.60%	2.40%	3.10%	7.00%	16.40%	18.40%	51.10%
I feel accepted by my parents as I am	3.00%	4.20%	6.80%	8.80%	17.60%	20.40%	39.10%
I believe that my opinion is important to my family	11.70%	10.70%	17.60%	17.30%	14.70%	12.80%	15.20%
I trust my parents	8.60%	8.70%	18.70%	15.20%	14.70%	14.20%	19.90%
I believe that my parents trust me	1.80%	2.10%	4.40%	6.50%	21.20%	23.10%	40.90%
My parents have given me the freedom to choose	1.30%	2.50%	4.30%	6.80%	17.30%	24.50%	43.20%
My parents are satisfied with my achievements	11.40%	12.10%	22.30%	18.90%	15.20%	9.90%	10.20%

Figure S2. a. Distribution of responses for the dimensions of roots of perfectionism: Relationships with family.

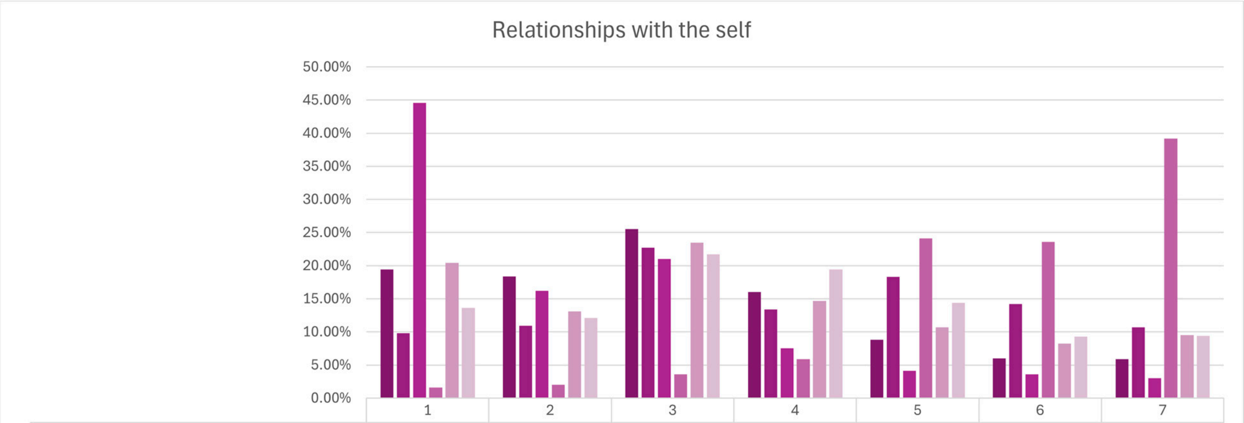

	1	2	3	4	5	6	7
When I make a mistake, I believe that I am at fault	19.40%	18.40%	25.50%	16.00%	8.80%	6.00%	5.90%
No matter what I do, I feel inadequate	9.80%	10.90%	22.70%	13.40%	18.30%	14.20%	10.70%
I am afraid of failing	44.60%	16.20%	21.00%	7.50%	4.10%	3.60%	3.00%
I belief I am worthy as a person	1.60%	2.00%	3.60%	5.90%	24.10%	23.60%	39.20%
When I make a mistake, I feel like a failure	20.40%	13.10%	23.50%	14.70%	10.70%	8.20%	9.50%
I feel like I don't have much to be proud of	13.60%	12.10%	21.70%	19.40%	14.40%	9.30%	9.40%

Figure S2. b. Distribution of responses for the dimensions of roots of perfectionism: Relationships with the self.

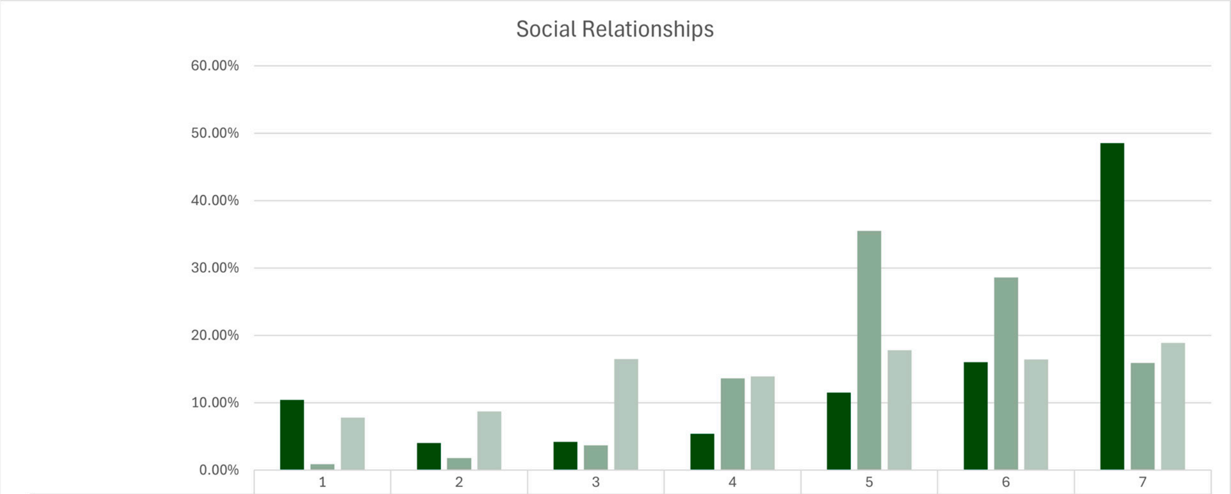

	1	2	3	4	5	6	7
I feel accepted by others as I am	10.40%	4.00%	4.20%	5.40%	11.50%	16.00%	48.50%
I feel trusted by others	0.90%	1.80%	3.70%	13.60%	35.50%	28.60%	15.90%
I feel I can trust others	7.80%	8.70%	16.50%	13.90%	17.80%	16.40%	18.90%

Figure S2. c Distribution of responses for the dimensions of roots of perfectionism: Social relationships.

Table S1. Items of the Multidimensional Perfectionism Scale Revised (MPS-R).

Code	Item
MPS1	When I am working on something, I cannot relax until it is perfect
MPS2	I find it difficult to meet others' expectations of me.
MPS3	One of my goals is to be perfect in everything I do.
MPS4	Everything that others do must be of top-notch quality.
MPS5	I feel that people are too demanding of me.
MPS6	It makes me uneasy to see an error in my work.
MPS7	I cannot stand to see people close to me make mistakes.
MPS8	The people around me expect me to succeed at everything I do
MPS9	I do not have to be the best at whatever I am doing.
MPS10	I have high expectations for the people who are important to me.
MPS11	My family expects me to be perfect.
MPS12	I set very high standards for myself
MPS13	The people who matter to me should never let me down
MPS14	Success means that I work even harder to please others

Table S2. Item of the Roots scale.

Code	Item
Roots1	I perceived myself as receiving love from my parents.
Roots2	When I make a mistake, I believe that I am at fault.
Roots3	I feel accepted by my parents as I am.
Roots4	I believe that my opinion is important to my family.
Roots5	No matter what I do, I feel inadequate.
Roots6	I trust my parents.
Roots7	I feel accepted by others as I am.
Roots8	I believe that my parents trust me.
Roots9	I am afraid of failing.
Roots10	I feel trusted by others.
Roots11	My parents have given me the freedom to choose in important matters.
Roots12	I belief I am worthy as a person, regardless of my mistakes.
Roots13	My parents are satisfied with my achievements based on my efforts.
Roots14	When I make a mistake, I feel like a failure.
Roots15	I feel like I don't have much to be proud of.
Roots16	I feel I can trust others.

Robustness Check: Comparison of Categorical and Continuous Analyses

To assess the potential influence of categorizing Socially Prescribed Perfectionism (SPP) on the study's conclusions, a sensitivity analysis was conducted comparing results derived from group-based analyses (low, moderate, and high SPP) with those obtained from multivariable linear regression treating SPP as a continuous variable. The results showed a high degree of convergence across both analytical approaches (Table S3). Specifically, sociodemographic associations observed in the categorical analyses—such as significant effects of age and religious category—were independently replicated in the continuous regression model ($p < 0.05$). In addition, the continuous approach offered greater analytical precision by quantifying the incremental variance explained by the Roots subscales ($\Delta R^2 = 0.255$). Taken together, these findings indicate that the use of categorical groupings, while intended to enhance clinical and educational interpretability, did not bias the results or obscure the primary relationships in the data.

Table S3. Sensitivity Analysis: Comparison of Results between Categorical (Group-based) and Continuous (Regression-based) Approaches for Socially Prescribed Perfectionism (SPP).

Factor	Categorical Analysis (Non-parametric Comparisons)	Continuous Analysis (Hierarchical Regression - Model 2)	Consistency / Robustness Result
**Age**	Higher SPP in younger groups; gradual decrease across age categories.	Significant negative predictor (β = -0.034, *p* =0 .045).	**Consistent**: Both methods identify age as a significant factor in SPP.
**Sex**	Females reported slightly higher SPP levels than males.	Non-significant after controlling for Roots (β = 0.021, *p* = 0.216).	**Refined**: Categorical differences are explained by relational/self roots in the multivariable model.
**Religious Category**	Modest differences primarily at the extremes of the distribution.	Significant negative predictor (β = -0.085, *p* <0.001).	**Consistent**: Significance is maintained and more precisely quantified in the continuous model.
**Roots: Family Relationships**	Negative correlation with maladaptive perfectionism and demandingness.	Significant unique negative predictor (β = -0.260, *p* < 0.001).	**Consistent**: Robust negative association with perceived external pressure.
**Roots: Relationship with the Self**	Lowest scores on protective subscale associated with higher fear of failure.	Strongest significant unique negative predictor (β = -0.285, p < 0.001).	**Consistent**: Identified as the primary protective factor against SPP in both analyses.
**Roots: Social Relationships**	Generally positive but lower trust associated with higher perceived demands.	Significant unique negative predictor (β = -0.096, *p* < 0.001).	**Consistent**: Confirms the relevance of social trust as a predictor.
**SOP & OOP**	Associated with the general perfectionism profile.	Significant positive predictors (*β*_{SOP} = 0.194, *β*_{OOP} = 0.137; *p* < 0.001).	**Consistent**: Demonstrates that SPP is related to, but distinct from, other dimensions.

Table S4. Effect sizes for group comparisons of Socially Prescribed Perfectionism (SPP).

Variable	Statistical Test	Effect Size Type	Value	*p*-value	Interpretation
**Sex**	Mann-Whitney U	Rank-Biserial Correlation (*r*)	0.130	< 0.001	Small
**Age Category**	Kruskal-Wallis	Epsilon-squared (ε^2)	0.008	< 0.001	Small
**Area of Study**	Kruskal-Wallis	Epsilon-squared ($\varepsilon 2$)	0.003	0.042	Negligible
**Level of Study**	Mann-Whitney U	Rank-Biserial Correlation (*r*)	0.001	0.975	Non-significant
**Private University**	Mann-Whitney U	Rank-Biserial Correlation (*r*)	0.003	0.925	Non-significant
**Offsite Status**	Mann-Whitney U	Rank-Biserial Correlation (*r*)	0.036	0.203	Non-significant
**Scholarship/Merit**	Mann-Whitney U	Rank-Biserial Correlation (*r*)	0.059	0.086	Non-significant
**Religious Practice**	Mann-Whitney U	Rank-Biserial Correlation (*r*)	0.008	0.781	Non-significant

Note. *Effect sizes were interpreted according to conventional benchmarks: for r, small ≈ 0.10 and medium ≈ 0.30; for* ε^2*, small ≈ 0.01 and medium ≈ 0.06.*
